# Sustained Remission in a Patient With Depression, Chronic Insomnia, and Ischemic Heart Disease Using a Combination of Agomelatine and Mirtazapine: A Case Report

**DOI:** 10.7759/cureus.106520

**Published:** 2026-04-06

**Authors:** Mohamed H Ahmed, Mohammed A Alhaj, Sadiyah Khan, Ahmed Hamid

**Affiliations:** 1 Psychiatry, Sheikh Khalifa Medical City Ajman, Ajman, ARE; 2 Medicine, Gulf Medical University, Dubai, ARE; 3 Internal Medicine, Sheikh Khalifa Medical City Ajman, Ajman, ARE

**Keywords:** agomelatine, cardiovascular risk, diabetes, hypothyroidism, insomnia, ischemic heart disease, major depressive disorder, metabolic diseases, mirtazapine

## Abstract

Agomelatine and mirtazapine are antidepressants with complementary pharmacological profiles. However, this combination is rarely reported in clinical practice. We report a case of a 54-year-old man with ischemic heart disease, diabetes, and hypothyroidism, who achieved early full remission of major depressive disorder and chronic insomnia following treatment with agomelatine and mirtazapine. By the four-week follow-up, the patient reported sleeping 8-9 hours/night with normalized mood and cognitive function. He sustained full remission for over three months, with no adverse effects. Serial lab investigations and ECGs remained stable throughout. No arrhythmic events or QTc prolongation were observed during follow-up.

While not commonly reported together in routine care, the combination was well tolerated and appeared to support both mood improvement and sleep restoration, without notable side effects, despite the presence of multiple cardiovascular and metabolic comorbidities.

This case adds to the expanding literature and suggests that this combination may be a useful off-label option for select patients with depression and insomnia who are at risk of cardiovascular or metabolic complications.

## Introduction

Major depressive disorder (MDD) is one of the most prevalent psychiatric disorders, characterized by low mood, fatigue, anhedonia, emotional/cognitive issues, and disrupted sleep patterns [1]. Insomnia is among the most common sleep disorders, characterized by difficulties such as falling or staying asleep [2]. MDD with comorbid insomnia is common in patients with chronic illnesses [3,4].

The coexistence of mood and sleep disturbances in individuals with cardiovascular or metabolic conditions is clinically significant, as standard antidepressant therapies may be limited due to a higher risk of side effects. Therefore, the pharmacological approach in these individuals must balance efficacy while maintaining metabolic neutrality and cardiac safety [5].

Agomelatine is a melatonergic agent demonstrating MT1 and MT2 receptor agonist activity and additionally acting as an antagonist of serotonin 5-HT₂C receptors [6]. Mirtazapine, a tetracyclic antidepressant, also classified as a noradrenergic and specific serotonergic antidepressant (NaSSA), promotes the release of serotonin and norepinephrine by inhibiting central presynaptic α2-adrenergic receptors. It also acts as an antagonist of histamine receptors (H1) and serotonergic 5-HT₂A and 5-HT₂C, and 5-HT₃ receptors.

Agomelatine is recognized for enhancing sleep quality through its action on melatonin receptors and is generally well tolerated with a low risk of serotonin syndrome, sexual dysfunction, and discontinuation syndrome [7]. Mirtazapine, in contrast to tricyclic antidepressants, has not demonstrated any notable adverse cardiovascular effects, demonstrating a reassuring cardiovascular safety profile, with minimal effects on cardiac conduction, QT interval, and arrhythmic risk even at dosages 22 times higher than the recommended maximum [8].

This combination is reported to have a relatively low serotonergic burden, reducing the likelihood of serotonin syndrome and QT prolongation, which are important considerations in patients with ischemic heart disease. Their combined sleep-regulating and mood-enhancing effects may also provide dual therapeutic benefits without exacerbating underlying health conditions [9-11]. Despite the complementary pharmacological profiles of agomelatine and mirtazapine, clinical reports describing their combined use remain scarce, and available evidence is largely theoretical or extrapolated from individual drug effects.

This case demonstrates the successful outcome of this combination in a patient with significant cardiac and metabolic comorbidities, in whom commonly used antidepressants such as selective serotonin reuptake inhibitors (SSRIs) and serotonin and norepinephrine reuptake inhibitors (SNRIs) may be limited by concerns related to tolerability, sleep disturbance, or cardiovascular effects, making alternative pharmacological strategies clinically relevant despite limited direct evidence.

## Case presentation

We report the case of a 54-year-old Egyptian male with a history of longstanding insomnia and low mood who presented with worsening symptoms over the past two years. His medical history was significant for ischemic heart disease with an ejection fraction of 30% and nine coronary stents, type 2 diabetes mellitus, hypothyroidism, and benign prostatic hyperplasia. At baseline, the patient presented with moderate-to-severe depressive symptoms including persistent low mood, anhedonia, fatigue, impaired concentration, and functional impairment. Insomnia was characterized by prolonged sleep latency and frequent awakenings. No standardized rating scales were used; assessment was based on serial clinical evaluation. Alternative treatment options were considered in the context of significant cardiovascular and metabolic comorbidities. The selected regimen was chosen to address both depressive symptoms and insomnia while minimizing serotonergic burden and potential cardiac or metabolic adverse effects.

He was started on agomelatine 25 mg and mirtazapine 30 mg nightly. The selected doses were chosen to optimize antidepressant efficacy while addressing insomnia, with consideration of tolerability and the patient’s cardiovascular and metabolic profile. At the two-week follow-up, he reported mild improvement in sleep and no adverse effects. By week four, his sleep had normalized to 8-9 hours per night, and his mood was euthymic. Remission was defined clinically as sustained normalization of mood, restoration of sleep, and return to baseline functional status. At the three-month follow-up, he remained in sustained remission, with normal laboratory results and electrocardiograms. Follow-up included regular clinical assessments, with laboratory and electrocardiographic monitoring performed at baseline and during treatment.

Investigations throughout the treatment period confirmed stable hepatic, renal, endocrine, and cardiac parameters (Table 1). No clinically significant changes in cardiac rhythm or QTc interval were observed. Hepatic parameters remained within normal limits throughout treatment. Concomitant medications remained stable, with no clinically significant interactions identified. The observed increase in triglyceride levels was consistent with the patient’s underlying metabolic profile and did not necessitate modification of psychiatric treatment.

**Table 1:** Laboratory results of the patient, with corresponding reference ranges. Note: Reference ranges adapted from American Board of Internal Medicine [12] and the National Kidney Foundation [13].

Parameter	Result	Interpretation	Reference range
Alanine aminotransferase	28–37 U/L	Normal hepatic profile	10–40 U/L
Albumin	40–41 g/L	Normal	35–55 g/L
Triglycerides	2.87 → 4.80 mmol/L	Moderate dyslipidemia	Optimal: <1.13 mmol/L; Normal: <1.70 mmol/L; Borderline-high: 1.70–2.25 mmol/L; High: 2.26–5.64 mmol/L; Very high: >5.64 mmol/L
Creatinine	95 µmol/L	Normal	Female: 44–97 µmol/L; Male: 62–115 µmol/L
Hemoglobin A1C	7.1–7.14%	Above target glycemic control	4.0%–5.6%
Thyroid-stimulating hormone	15.91 → 2.62 mU/L	Normalized with treatment	0.5–4.0 mU/L [12]
estimated glomerular filtration rate (eGFR)	76–82 mL/min	Stable renal function	>90 mL/min/1.73m² [13]

## Discussion

The combination of agomelatine and mirtazapine is rarely reported in clinical practice but is pharmacologically rational, especially in patients with significant medical comorbidities. Agomelatine helps regulate circadian rhythms without serotonergic overload [7,14], while mirtazapine improves sleep and appetite with minimal serotonergic and anticholinergic adverse effects [15-19]. Both agents carry a low risk of QTc prolongation or serotonin syndrome, making them more suitable for patients with ischemic heart disease, polypharmacy, or metabolic disorders [9-11]. Compared to tricyclic antidepressants and some SNRIs, which are associated with QT prolongation and arrhythmic risk, this combination may offer a lower cardiotoxic burden.

In our case, the patient achieved and maintained full remission over three months with no sedation, metabolic disturbance, or cardiac issues, demonstrating the regimen’s tolerability and potential clinical value in medically complex presentations. The observed improvement may reflect both pharmacological effects and non-specific factors such as the natural course of illness or placebo response.

This case contributes to the limited literature supporting the safety and efficacy of combining select antidepressants in high-risk populations. Limitations include the single-case design, relatively short follow-up duration, and the absence of standardized rating scales or objective sleep measures. This approach may be particularly relevant for patients with depression and insomnia in the context of cardiovascular or metabolic comorbidities. It further emphasizes the importance of tailoring antidepressant treatment to individual risk profiles, especially in patients for whom standard SSRIs or SNRIs may be inappropriate due to cardiovascular or metabolic concerns.

## Conclusions

The combination of agomelatine and mirtazapine may provide a useful therapeutic option for individuals diagnosed with both depression and insomnia, particularly those who also have elevated cardiovascular or metabolic risk. Together, these medications have a favorable side effect profile and may display synergistic effects, with mirtazapine providing noradrenergic and sedative effects and agomelatine contributing to circadian rhythm regulation and dopaminergic response. These characteristics make it a potentially appropriate choice for patients with complex health profiles.

Limited clinical data exist on this combination, and additional randomized controlled trials are necessary to confirm the safety and efficacy of this combination. However, evidence remains limited, and further controlled studies are required to confirm these findings.
